# Gene Expression Profiling during Early Acute Febrile Stage of Dengue Infection Can Predict the Disease Outcome

**DOI:** 10.1371/journal.pone.0007892

**Published:** 2009-11-19

**Authors:** Eduardo J. M. Nascimento, Ulisses Braga-Neto, Carlos E. Calzavara-Silva, Ana L. V. Gomes, Frederico G. C. Abath, Carlos A. A. Brito, Marli T. Cordeiro, Ana M. Silva, Cecilia Magalhães, Raoni Andrade, Laura H. V. G. Gil, Ernesto T. A. Marques

**Affiliations:** 1 Departamento de Virologia e Terapia Experimental, Centro de Pesquisas Aggeu Magalhães-CPqAM/FIOCRUZ, Recife, Pernambuco, Brazil; 2 Departamento de Imunologia do Centro de Pesquisas Aggeu Magalhães-CPqAM/FIOCRUZ, Recife, Pernambuco, Brazil; 3 Department of Electrical Engineering, Texas A&M University College Station, Texas, United States of America; 4 Departamento de Medicina Clínica, Universidade Federal de Pernambuco, Recife, Pernambuco, Brazil; 5 Hospital Esperança, Recife, Pernambuco, Brazil; 6 Laboratório Central de Saúde Pública, Secretaria de Saúde do Estado de Pernambuco, Recife, Pernambuco, Brazil; 7 Department of Medicine, Division of Infectious Disease, The Johns Hopkins University School of Medicine, Baltimore, Maryland, United States of America; 8 Department of Pharmacology and Molecular Sciences, The Johns Hopkins University School of Medicine, Baltimore, Maryland, United States of America; 9 Department of Infectious Diseases and Microbiology, University of Pittsburgh, Pittsburgh, Pennsylvania, United States of America; Singapore Immunology Network, Singapore

## Abstract

**Background:**

We report the detailed development of biomarkers to predict the clinical outcome under dengue infection. Transcriptional signatures from purified peripheral blood mononuclear cells were derived from whole-genome gene-expression microarray data, validated by quantitative PCR and tested in independent samples.

**Methodology/Principal Findings:**

The study was performed on patients of a well-characterized dengue cohort from Recife, Brazil. The samples analyzed were collected prospectively from acute febrile dengue patients who evolved with different degrees of disease severity: classic dengue fever or dengue hemorrhagic fever (DHF) samples were compared with similar samples from other non-dengue febrile illnesses. The DHF samples were collected 2–3 days before the presentation of the plasma leakage symptoms. Differentially-expressed genes were selected by univariate statistical tests as well as multivariate classification techniques. The results showed that at early stages of dengue infection, the genes involved in effector mechanisms of innate immune response presented a weaker activation on patients who later developed hemorrhagic fever, whereas the genes involved in apoptosis were expressed in higher levels.

**Conclusions/Significance:**

Some of the gene expression signatures displayed estimated accuracy rates of more than 95%, indicating that expression profiling with these signatures may provide a useful means of DHF prognosis at early stages of infection.

## Introduction

The dengue virus is a member of *Flaviviridae* family, genus *flavivirus* with four antigenically distinct serotypes (DENV-1 to DENV-4). Dengue virus infection is a global public health concern, with an estimated incidence of 50–100 million cases of dengue fever (DF), resulting in 500,000 clinical cases of life-threatening dengue hemorrhagic fever syndrome (DHF) and 24,000 deaths per year [1]. DHF is characterized by vasculopathy, which results in sudden plasma leakage that reduces the blood volume and may result in hypovolemic shock, known as dengue shock syndrome (DSS). There is no antiviral therapy to treat dengue infection, neither are there means to prevent the development of DHF.

During the acute febrile phase of infection, DF and DHF patients display a very similar clinical picture. However at defervescence (after 4 to 7 days of the beginning of the symptoms), DHF patients start to present signs of circulatory disturbance [2], which makes medical management a major challenge in endemic areas. This is especially true during outbreaks when dengue cases typically over saturates the capacity of all medical points-of-care, and results in shortage on the capacity to attend the regular demand for medical assistance causing major disruptions on the public health systems.

The World Health Organization [2] has established clinical criteria to define DHF cases, but the difficulties of both fulfilling these criteria and early identification of severe cases, make the clinical management of severe forms of the disease even a greater challenge [3], [4]. Therefore, a search for new tools to predict patient outcome is essential to facilitate the assessment of the need for medical interventions.

The current concept underlining DHF immunopathology relies on epidemiological evidence indicating an increased risk of developing DHF in secondary dengue infections. This concept led to the identification of biological mechanisms involving antibody mediated enhancement (ADE), mediated by non-neutralizing antibodies [5], [6], as well as exacerbated activation of cross-reactive T cell clones [7], [8], [9], [10], both acquired after primary infection. Some of the “markers” of dengue severity found on peripheral blood that were correlated with plasma leakage on DHF include several inflammatory cytokines, chemokines and adhesion molecules that are expressed at high levels [11], [12], [13], [14], [15], [16], [17], complement activation products [18], [19], increased frequency of activated cells [20], [21] and large number of cells undergoing apoptosis [9], [22]. All these findings have helped to advance the understanding of the immune mechanisms involved in the development of DHF but none of them were proven to be reliable or useful as biological markers for clinical use.

DNA microarrays have been used as a tool to identify “signature genes” and predict successfully, patient outcome for cancer [23], [24] as well as for bacterial and viral infections [25], [26]. Using this approach, some studies have shown that at the peak of the disease, several genes are differentially expressed in dengue patients presented with the more severe disease [27], [28]. Hence, we believe that an early gene expression signature differentiating the mild from the severe clinical outcomes of dengue could be a useful tool for developing biomarkers to predict clinical outcome, which will facilitate the clinical management of dengue infected patients.

Here we report the analysis of gene-expression microarray data of PBMC samples collected from DF and DHF patients during the febrile phase of the disease. These data were used as the basis for the development of reliable biomarkers to predict the clinical outcome of dengue infection.

## Methods

### Dengue Cohort Design and Strategy for Functional Genomic Studies

A cohort of acute febrile patients admitted on three hospitals in the city of Recife, state of Pernambuco, Brazil, was established and described elsewhere [29], [30]. Briefly, sequential blood samples were obtained at the day of admission, day 1, and at days 3, 5, 7, 15 and 30. Dengue cases (confirmed by either serology, RT-PCR or virus isolation) were clinically classified according to the WHO criteria into two classes: Dengue Fever (DF) and Dengue Hemorrhagic Fever (DHF) [31]. All participants signed an informed consent. This study, that included several methods suitable for studies related to dengue immunopathology including functional immunomics, was reviewed and approved by ethics committee of Brazilian Ministry of Health CONEP: 4909; Process n° 25000.119007/2002-03; CEP: 68/02. In addition, the Johns Hopkins IRB also reviewed this study as protocol JHM-IRB-3: 03-08-27-01.

The inclusion criterion for this functional genomic study was: All the subjects enrolled had to have at least three medical visits within the first two weeks of study enrollment. The dengue patients had to have confirmed acute dengue 3 infection based on RT-PCR/virus isolation and serology, be febrile at the time of the first hospital visit (temp. above 38.5°C) and with more than 10×10^6^ PBMCs available for microarray analysis collected at the first visit. Moreover, for DHF group, the samples must be collected prior the onset of circulatory disturbances (hematocrit and levels of serum albumin normal) and no signs of bleeding (tourniquet test negative). Samples had to have clear definition of the clinical outcome of either DF or DHF. A non-dengue group of patients (ND) consisting of individuals with febrile infection of unknown etiology with negative tests results for dengue by RT-PCR, virus isolation and serology after at least 3 samples collected within the first two weeks after enrollment. This group includes suspected dengue cases collected during the same period as the dengue cases, but for which dengue infection was not confirmed through either RT-PCR/virus isolation or serology in at least three blood samples collected at different days. The samples from DF, DHF and ND patients were matched to avoid spurious associations with patient age, gender, dengue infection history and days of symptoms between the groups.

The functional genomic studies were performed on total RNA extracted from PBMC purified from blood samples collected from febrile patients at the time of their first medical visit. The samples selected for this study were collected from 18 confirmed dengue 3, genotype III cases and 8 control samples (ND group). None of the DHF patients presented vasculopathy signs and symptoms at the time the samples used in the functional genomic characterization were collected. At the time of collection the patients referred approximately 5 days of disease and the absence of fever was reported two to three days after enrollment. Among the dengue confirmed cases, 8 patients were characterized as DF and 10 patients were classified as DHF (Table 1).

**Table 1:** Patients selected for functional genomic studies.

					Dengue diagnosis		
Patients	Clinical Diagnosis	Sex	Age	Days of Symptoms	IgM	IgG	PCR/virus isolation
P330	DF	F	40	4	Neg	Pos	Pos
P310	DF	F	30	3	Neg	Pos	Pos
P331	DF	M	45	6	Pos	Pos	Pos
P121	DF	M	53	4	Neg	Pos	Pos
P129	DF	F	29	5	Neg	Pos	Pos
P164	DF	M	27	1	Neg	Pos	Pos
P171	DF	M	44	5	Neg	Pos	Pos
P243	DF	F	23	7	Pos	Neg	Pos
P277	DHF	M	41	3	Neg	Pos	Pos
P307	DHF	F	41	8	Pos	Neg	Pos
P125	DHF	F	84	8	Pos	Pos	Pos
P128	DHF	F	26	7	Pos	Neg	Pos
P145	DHF	M	19	7	Pos	Neg	Pos
P165	DHF	F	22	4	Neg	Neg	Pos
P206	DHF	M	36	8	Pos	Pos	Pos
P235	DHF	F	35	5	Pos	Pos	Pos
P102	DHF	F	21	7	Pos	Neg	Pos
P111	DHF	F	21	5	Pos	Neg	Pos
P317	ND	M	41	4	Neg	Neg	Neg
P237	ND	M	25	8	Neg	Neg	Neg
P239	ND	M	47	4	Neg	Neg	Neg
P251	ND	M	54	4	Neg	Neg	Neg
P195	ND	F	23	6	Neg	Neg	Neg
P199	ND	F	19	6	Neg	Neg	Neg
P216	ND	F	30	2	Neg	Neg	Neg
P269	ND	F	64	4	Neg	Neg	Neg
**P430**	DHF	F	25	8	Neg	Pos	Neg
**P586**	DHF	F	16	5	Pos	Pos	Pos
**P557**	DHF	F	76	9	Pos	Neg	Pos
**P549**	DHF	F	10	11	Pos	Pos	Neg
**P543**	DHF	F	29	11	Pos	Pos	Neg
**P586**	DHF	F	16	7	Pos	Pos	Pos
**P414**	DHF	M	34	5	Neg	-	-
**P305**	DHF	M	35	6	Pos	Pos	Pos
**P677**	DF	F	69	8	Pos	Pos	Neg
**P659**	DF	F	32	8	Pos	Pos	Neg
**P650**	DF	F	58	8	Neg	Pos	Pos
**P633**	DF	F	39	8	Pos	Neg	Neg
**P634**	DF	F	27	8	Pos	Pos	Pos
**P620**	DF	M	62	8	Pos	Pos	Neg
**P600**	DF	M	52	8	Neg	Pos	Neg
**P588**	DF	M	26	5	Neg	Neg	Pos
**P310**	DF	F	30	3	Neg	Pos	Pos

**In bold**: samples used exclusively in the qPCR assays. DF: Dengue Fever; DHF: Dengue hemorrhagic fever; ND: Non-Dengue; M: male; F: female; Pos: positive; Neg: negative; -: No information.

### Sample Processing for Genechip Hybridization

Blood samples from patients enrolled in this study were collected in heparin vacutainer tubes (BD Vacutainer) and within 2 hours from the collection, PBMC samples were separated by gradient density using Ficoll-Paque (Amersham Biosciences) and cryopreserved in 10% (v/v) Dimethyl sulfoxide (DMSO; Sigma-Aldrich) in inactivated fetal bovine sera (FBS; Hyclone). Four million frozen cells were thawed and immediately lysed with Trizol (Invitrogen) for total RNA extraction through chloroform extraction and isopropyl alcohol precipitation following manufacturer's protocol [gene expression using either fresh or frozen PBMC were compared and shown to be similar, (data not shown)]. The total RNA was purified by using the RNeasy MinElute Cleanup Kit (Qiagen) according to the manufacturer protocol and quantified by spectrophotometry at 260 nm and 280 nm (UV spectrum). Total RNA was used for cRNA synthesis through two-cycle target labeling kit (Affymetrix) according to the manual manufacturer. Briefly, RNA isolated from the PBMC was biotin-labeled and hybridized to human oligonucleotide microarrays (Affymetrix) by using a two-cycle methods of cDNA synthesis as follow. On the first cycle, first-strand cDNA was prepared by using a T7-(dT) primer and Superscript II reverse transcriptase (Invitrogen) from 10 to 100 ng of cellular RNA. Second strand synthesis was performed by using E. coli DNA polymerase I and the double-stranded cDNA was used for in vitro transcription (IVT) for cRNA amplification by using Megascript T7 kit (Ambion). The product of this first cycle of reaction (cRNA) was used for reverse transcription for synthesis of first and second strands of cDNA as described for the first cycle. This cDNA was used for IVT for synthesis of biotin-labeled cRNA, which was fragmented and sent to Microarray Core Facility at The Johns Hopkins University for target hybridization to Human Genome U133 Plus 2.0 DNA Chip (Affymetrix), staining and scanning.

### Microarray Data Quality Control

The quality of the microarray data was assessed using several criteria: visual inspection, noise/efficiency measurements, spike-in controls, housekeeping gene expression, and RNA degradation plots. Visual inspection based on the high-resolution. DAT files did not reveal smudges or streaks, and the B2-oligo probes (e.g., chessboard patterns around edges and name of array) were also visible. Noise/Efficiency measurements made by the Affymetrix MAS 5.0 software that can be used to evaluate the quality of the arrays are displayed in the Supplement material S1. Noise Q factors, background, scaling factors, and the percentages of present calls were similar across all arrays. Average background values were below 100 for 20 of the 26 microarrays; scaling factors were within three folds of each other, for 25 of the 26 microarrays; and the percentages of present calls were around 40% or higher for 25 of the 26 arrays. None of the six arrays that presented high background had a rate of present calls significantly below 40%. All 3′-probe sets and middle probe sets for all 4 spike-in poly-A control genes (*dap, thr, phe, lys*) were called present in all arrays, as were all probe sets for the four prokaryotic hybridization control genes (*bioB*, *bioC*, *bioD*, *cre*) and the human housekeeping genes GAPDH (human glyceraldehyde-3-phosphate dehydrogenase) and Beta-Actin. Furthermore, RNA degradation plots showing the average intensity of probes indicate an acceptable levels in all probe sets displayed (see in Supplement material S2).

### Quantitative Real Time PCR for Microarray Validation

Four DF/DHF differentially expressed genes (MT2A, PSMB9, C3aR1 and HLA-F) were selected for quantification by quantitative real time PCR (qPCR). Genes were amplified and detected using TaqMan® gene expression assays with primers and probes commercially designed (Applied Biosystems). RNA extraction was performed according to the manufacturer's manual for the RNeasy Kit (Qiagen). Total RNA was reverse transcribed to cDNA using SuperScript III First-strand Synthesis System for qPCR (Invitrogen) using random hexamer primers according to the manufacturer's instructions. Quantitative real time PCR was carried out on the ABI PRISM 7500 device (Applied Biosystems). Beta-Actin was selected as endogenous control and all data were normalized against it. The reactions were performed in triplicates and included 2 µl of cDNA, primers (20 µM each) and 6.25 µM of the specific probe or commercially pre-designed Gene Expression Assay Mix (Applied Biosystems), Human Beta-Actin (Applied Biosystems), TaqMan Universal PCR Master Mix (Applied Biosystems) and water added to a final volume of 25 µl. Triplicates of non-template controls (NTC) were included each time qPCR was undertaken. Cycle conditions were as follows: after an initial 2-min hold at 50°C and 10 minutes at 95°C, the samples were cycled 40 times at 95°C for 15 sec and 60°C for 1 min. Baseline and threshold for cycle threshold (Ct) calculation were set manually with Sequence Detection Software version 1.4. The efficiency of amplification (E) of a target molecule was calculated from the slope of the standard curve (plot of Ct versus the negative log10 concentration of the target) derived from the slopes (E = 10̂(−1/Slope)−1). For relative calculation the Delta Ct method was used [ABI PRISM® 7000 Sequence Detection System and Applied Biosystems 7500 Real-Time PCR System - User Bulletin, Applied Biosystems] once all assays met the amplification efficiency criteria of 100%±10% [Application Note 127AP05-02]. Samples used in the qPCR assays are described on Table 1 (samples of ND patients and DHF patients number 105 and 112 were not used).

### Statistical Analysis

Patient data quality-control, statistical analysis, and plotting were carried out using Affymetrix MAS 5.0 software [32] and the open source R statistical package, version 2.5.0 [33], and add-on libraries, in particular the BioConductor library, version 1.16 [34]. Dendrograms and MDS plots were produced with the R functions “hclust” and “isoMDS”, respectively, whereas heatmaps were obtained with the functions “hset.emap” and “heatmap”. All microarray data is MIAME compliant and the raw data has been deposited in a MIAME compliant database as accession number # GSE18090 and it is available at http://www.ncbi.nlm.nih.gov/geo/query/acc.cgi?token=lpofdqcuugmwsng&acc=GSE18090. P-values corresponding to two-tailed Welch's two-sample t-tests were obtained with the function “t.test”. Functional category overrepresentation analysis was performed with the performed with the EASE program (Expression Analysis Systematic Explorer) at the DAVID Bioinformatics resource website (http://david.abcc.ncifcrf.gov/) [35]. The Linear Discriminant Analysis classification method implemented by directly estimating the sample means and covariance matrices for each diagnostic class [36]. Classification accuracy was estimated by the method of bolstered resubstitution [37], which displays good properties for gene-set selection in small-sample situations [38], [39]

## Results

### Detection and Variance Filters

After careful quality control analyses of each genechip, Affymetrix MAS 5.0 software was used to analyze the data. From 54,675 gene transcripts on each of the 26 arrays, 12,299 were called present (p-value <0.04) on all arrays, whereas 20,365 were not called present on any of the arrays. In order to retain only promising genes and enable significant statistical analysis, we chose to analyze the genes that were called present on at least 24 of the 26 arrays used in this study. This filter resulted in a total of 15,848 genes and among those, 3,549 genes (15,848−12,299 = 3,549) that were called absent on one or two arrays. Subsequently we applied a variance filter to eliminate constitutive or housekeeping genes, which retained the top 1/8 gene in variance out of 15,848 previous genes, which resulted in 1981 “best” genes.

### Exploratory Analysis

**Figure 1:**
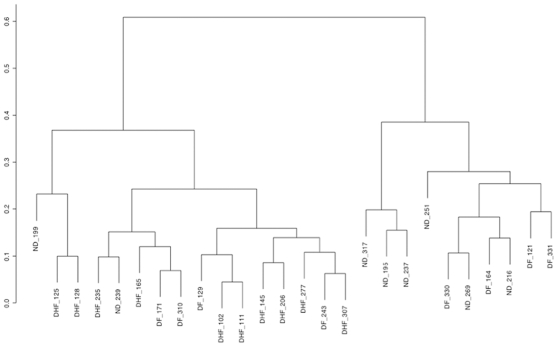
Dendrogram of hierarchical clustering considering 1981 genes.

**Figure 2:**
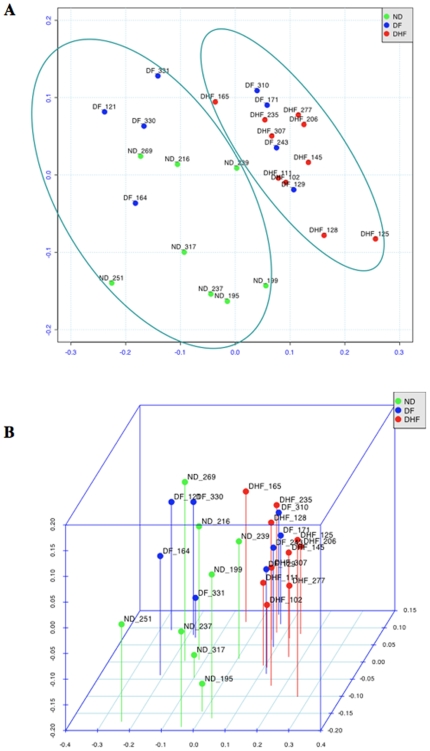
Multidimensional scaling (MDS) plots for the 1981 selected genes. (A) 2-D MDS plot. (B) 3-D MDS plot. The elipses in (B) depmarcate two major groups of samples (please see text).

Figure 1 displays the dendrogram for the hierarchical clustering of the 26 samples according to the expression of the 1981 genes selected at the preprocessing stage. Average linkage and Pearson correlation of log-transformed expression values were employed. We can see that the samples fell into two major groups; the one on the right contains only ND samples, and half (4) of the DF samples, while the group on the left contains all the DHF samples, a couple of ND samples (including one outlier, ND_199), and the other half of the DF samples. This agrees with intuition, since the two most different groups should be ND and DHF, with the DF samples forming an intermediary group. This is confirmed by the 2-D and 3-D multidimensional scaling (MDS) plots for the 1981 selected genes, displayed in Figures 2A and 2B. The dissimilarity measure used was 1−r, where r is Pearson correlation of log-transformed values. Arrays were colored according to diagnostic class. It can be seen that the DHF samples constitute a tighter cluster than the ND samples, as expected since ND samples were obtained from patient with fever of unknown etiology, whereas the DF samples could be divided in two groups, one similar to the DHF samples, and another not similar to DHF, and in fact closer in expression to some of the ND samples (these are the two groups marked in Figure 2A). The small stress values (13.52% in the 2-D case and 7.32% in the 3-D case), particularly in the 3D case, mean that the underlying structure of the data is intrinsically a low-dimensional one, which indicates that a small number of signature genes might be able to account for the discriminatory content in the data.

### Differential Expression Analysis

**Figure 3:**
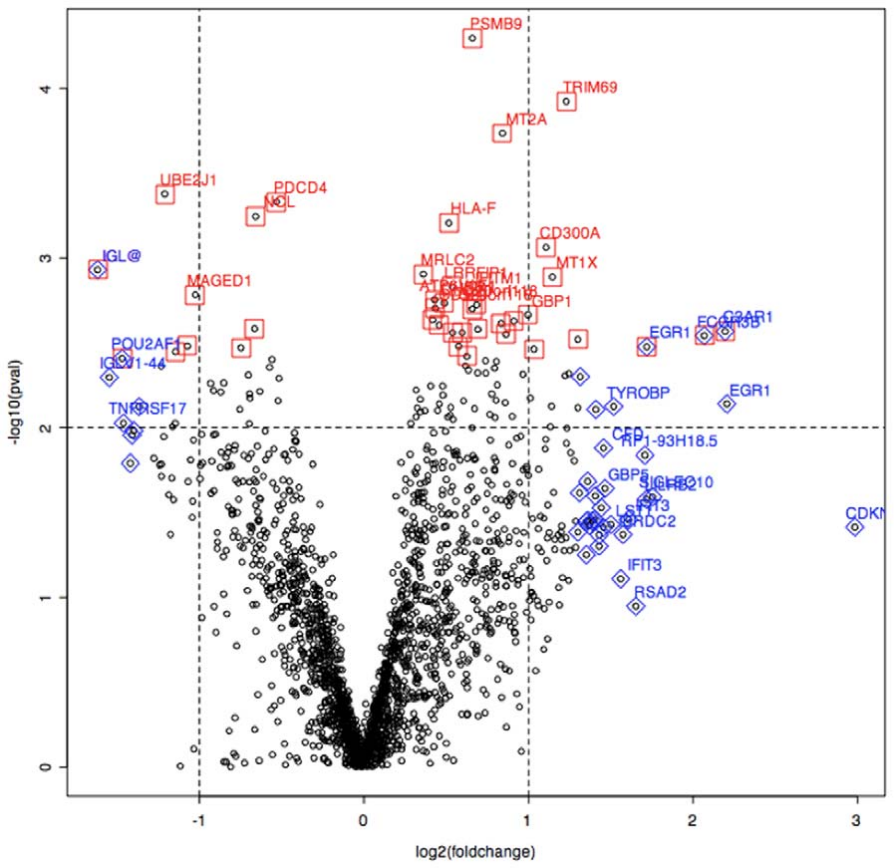
Volcano plot showing p-values correlated to fold changes in DF vs DHF samples. The genes highlighted in red and blue represent the top 40 genes according to the p-values and fold change respectively.

**Figure 4:**
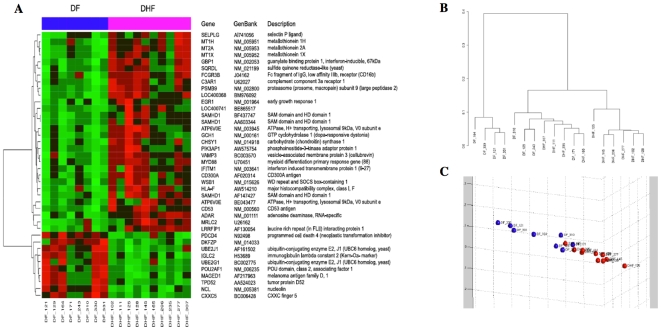
Top 40 genes differentially expressed between DF and DHF samples. (A) Expression heatmap with GenBank annotations. (B) Hierarchical clustering dendrogram (only top 40 genes). (C) 3-D MDS plot (only top 40 genes).

Statistical tests of differences between means (Welch two-sample t-tests) were performed to show the most differentially expressed mRNAs among the diagnostic groups. We considered four comparisons among groups: DF vs. DHF samples; Dengue (DF+DHF) vs. ND samples; DF vs. ND samples; DHF vs. ND samples. The first comparison corresponds to the 18 samples that come from dengue patients and is the most important comparison for our purposes, as it discriminates between the severe hemorrhagic form of dengue and the benign one. Important genes used to characterize these two clinical outcomes were gathered on Table 2. The “volcano plot” on Figure 3 and the correspondent gene list on Table 3, show that p-values were well correlated to fold changes in DF vs. DHF samples as well as for the other comparisons (Supplement material S3). The largest differences were observed in the comparison of DHF vs. ND, as expected, followed by differences in (DHF+DF) vs. ND and DF vs. ND. The critical differences in gene expression between DF and DHF are the least pronounced. The top 40 genes with the most significant p-values among the 1981 genes previously selected, for each of the four comparisons, along with fold change values, average signal intensity, and annotation from the DAVID Bioinformatics Database at NIAID/NIH (http://david.abcc.ncifcrf.gov/) can be accessed in the Supplement material S4. Figure 4 shows the heatmap expression for the 40 top genes that discriminate the DF and DHF samples, according to the Welch t-test criterion, as well as the dendrogram of hierarch clustering and MDS plot for the 40 top DF-DHF discriminatory genes. Table 4 displays the results of functional overabundance analysis of the list of top 40 DF-DHF discriminatory genes performed with the EASE program (Expression Analysis Systematic Explorer) at the DAVID Bioinformatics resource website. The EASE analysis indicated the enrichment of certain categories of genes. Five categories presented significant scores (p<0.05) after Bonferroni correction for multiple tests, including the ones involved on immune and defense responses, response to biotic stimulus, copper/cadmium binding and copper ion homeostasis. The EASE results for all 4 comparisons can be accessed in the Supplement material S5.

**Table 2:** List of genes differentially expressed in DF patients in relation to DHF patients based on the lowest *p* and highest fold change values.

Symbol	Gene Title	p-value	Fold-change	Average signal
RNF36	ring finger protein 36	0.000119	2.3447	6.7868
UBE2J1	ubiquitin-conjugating enzyme E2, J1 (UBC6 homolog, yeast)	0.000419	−2.3147	7.3763
CD300A	CD300a molecule	0.000864	2.1544	7.079
IGL@	Immunoglobulin lambda locus	0.001172	−3.0718	6.3888
MT1X	metallothionein 1X	0.001294	2.2122	7.5485
MAGED1	melanoma antigen family D, 1	0.001644	−2.0342	7.0617
GBP1	guanylate binding protein 1, interferon-inducible, 67 kDa///guanylate binding protein 1, interferon-inducible, 67 kDa	0.00216	1.9967	7.8344
VAMP3	vesicle-associated membrane protein 3 (cellubrevin)	0.002351	1.8814	7.1191
C3AR1	complement component 3a receptor 1	0.002707	4.5817	6.0814
MYD88	myeloid differentiation primary response gene (88)	0.002823	1.8194	8.0611
FCGR3B	Fc fragment of IgG, low affinity IIIb, receptor (CD16b)	0.002868	4.1952	6.1543
SAMHD1	SAM domain and HD domain 1	0.003019	2.4624	5.7873
UBE2G1	ubiquitin-conjugating enzyme E2G 1 (UBC7 homolog, yeast)	0.003307	−2.1036	6.9206
EGR1	early growth response 1	0.00334	3.2916	6.1035
MT1H	metallothionein 1H	0.003445	2.0475	6.755
TPD52	tumor protein D52	0.003567	−2.2114	7.0498
POU2AF1	POU domain, class 2, associating factor 1	0.00391	−2.7707	8.0521
PPAPDC1B	Phosphatidic acid phosphatase type 2 domain containing 1B	0.004347	−2.3491	7.075
RP1-93H18.5	hypothetical protein LOC441168	0.004813	2.3726	8.0818
SIDT2	SID1 transmembrane family, member 2	0.00485	1.9548	8.3433
GBP1	guanylate binding protein 1, interferon-inducible, 67 kDa///guanylate binding protein 1, interferon-inducible, 67 kDa	0.004938	2.3334	7.0136
GBP1	guanylate binding protein 1, interferon-inducible, 67 kDa	0.005006	2.4888	7.5899
LOC96610///IGL@	Hypothetical protein similar to KIAA0187 gene product///Immunoglobulin lambda locus	0.005057	−2.9244	7.0226
HSP90B1	heat shock protein 90 kDa beta (Grp94), member 1	0.005488	−1.9074	8.2753
CRR9	cisplatin resistance related protein CRR9p	0.006054	−1.8843	7.3673
NDUFB6	NADH dehydrogenase (ubiquinone) 1 beta subcomplex, 6, 17 kDa	0.006174	−1.828	7.2738
RNASET2	ribonuclease T2	0.006686	2.0165	7.819
GBP2	guanylate binding protein 2, interferon-inducible///guanylate binding protein 2, interferon-inducible	0.007125	1.8139	7.6419
EGR1	Early growth response 1	0.007242	4.6155	6.0488
CD97	CD97 molecule	0.007485	2.2044	6.8153
TYROBP	TYRO protein tyrosine kinase binding protein	0.007507	2.8639	7.711
CTA-246H3.1	similar to omega protein	0.007524	−2.578	8.6364
SERPINA1	serpin peptidase inhibitor, clade A (alpha-1 antiproteinase, antitrypsin), member 1	0.00763	2.463	7.455
FGL2	fibrinogen-like 2	0.007811	2.6573	7.5789
TUBA3	tubulin, alpha 3	0.008598	1.8686	8.6244
TNFRSF17	tumor necrosis factor receptor superfamily, member 17	0.009406	−2.7543	7.2692
GMNN	geminin, DNA replication inhibitor	0.009426	−2.2142	7.1972
SMC4L1	SMC4 structural maintenance of chromosomes 4-like 1 (yeast)	0.009866	−2.2396	6.6128
FGR	Gardner-Rasheed feline sarcoma viral (v-fgr) oncogene homolog	0.009879	2.1941	7.2555
TNFSF13///TNFSF12-TNFSF13	tumor necrosis factor (ligand) superfamily, member 13///tumor necrosis factor (ligand) superfamily, member 12-member 13	0.010068	2.352	6.3382
IGL@	Immunoglobulin lambda locus	0.010428	−2.6368	6.334
GNAS	GNAS complex locus	0.010903	−1.8682	8.2788
UAP1	UDP-N-acteylglucosamine pyrophosphorylase 1	0.010949	−1.9816	6.8578
ARMET	arginine-rich, mutated in early stage tumors	0.011018	−1.8831	7.4254
---	Immunoglobulin kappa light chain (IGKV) mRNA variable region, joining region, and constant region///Immunoglobulin kappa light chain (IGKV) mRNA variable region, joining region, and constant region	0.011066	−2.6563	7.408
HLA-DPA1	major histocompatibility complex, class II, DP alpha 1	0.011235	1.9378	7.2184
IGKV1D-13///LOC649876	immunoglobulin kappa variable 1D-13///similar to Ig kappa chain V-I region HK102 precursor	0.011292	−2.3235	7.5191
PYCARD	PYD and CARD domain containing	0.011531	1.904	7.3968
KIAA1505	KIAA1505 protein	0.011735	2.152	7.5726
SEC11L3	SEC11-like 3 (S. cerevisiae)	0.011854	−2.0503	6.9297
CX3CR1	chemokine (C-X3-C motif) receptor 1	0.012371	2.3472	7.8999
MT1E	metallothionein 1E (functional)	0.012654	1.8414	6.5549
CFD	complement factor D (adipsin)	0.013175	2.7446	6.5182
C11orf75	chromosome 11 open reading frame 75	0.013342	2.1468	6.628
SRPRB	signal recognition particle receptor, B subunit	0.01398	−1.8251	7.2025
SAMD9L	sterile alpha motif domain containing 9-like	0.01406	2.3893	7.1224
RP1-93H18.5	hypothetical protein LOC441168	0.014525	3.2665	6.6864
PACAP	proapoptotic caspase adaptor protein	0.015153	−2.4229	7.0357
KIAA0746	KIAA0746 protein	0.015464	−2.0754	7.0611
HLA-DPB1	major histocompatibility complex, class II, DP beta 1	0.015645	2.4265	7.8066
PRDX4	peroxiredoxin 4	0.01571	−1.8982	7.3706
---	Transcribed locus	0.016136	−1.9733	6.7522
LOC652745	similar to Ig kappa chain V-I region Walker precursor	0.016187	−2.6756	7.035
TCF7L2	transcription factor 7-like 2 (T-cell specific, HMG-box)	0.016262	1.8697	8.0753
---	Immunoglobulin kappa chain, V-region (SPK.3)	0.01639	−2.3461	6.3625
PACAP	proapoptotic caspase adaptor protein	0.016535	−2.367	7.8079
ITM2C	integral membrane protein 2C///integral membrane protein 2C	0.016602	−1.9904	6.6164
C9orf19	chromosome 9 open reading frame 19	0.0169	1.9849	6.9941
LRRC59	leucine rich repeat containing 59	0.017101	−1.8304	7.1703
S100A9	S100 calcium binding protein A9 (calgranulin B)	0.017515	2.0885	8.328
SPTLC2	serine palmitoyltransferase, long chain base subunit 2	0.017518	1.9835	7.0682
PARP12	poly (ADP-ribose) polymerase family, member 12	0.017657	1.9566	7.7735
S100A6	S100 calcium binding protein A6 (calcyclin)	0.019028	2.0002	7.2831
*PSMB9*	proteasome (prosome, macropain) subunit, beta type, 9 (large multifunctional peptidase 2)	0.00005	1.5788	8.4562
*MT2A*	metallothionein 2A	0.000184	1.7931	8.322
*PDCD4*	programmed cell death 4 (neoplastic transformation inhibitor)	0.000465	−1.4445	7.5556
*NCL*	nucleolin	0.000569	−1.5782	7.7589
*HLA-F*	major histocompatibility complex, class I, F	0.000621	1.43	8.4362
*MRLC2*	myosin regulatory light chain MRLC2	0.001244	1.2847	8.6979
*LRRFIP1*	leucine rich repeat (in FLII) interacting protein 1	0.001476	1.4494	8.7862
*IFITM1*	interferon induced transmembrane protein 1 (9–27)	0.001576	1.5969	8.6038
*ATP6V0E*	ATPase, H+ transporting, lysosomal 9 kDa, V0 subunit e	0.00176	1.3477	8.9288
*CHSY1*	carbohydrate (chondroitin) synthase 1	0.001841	1.4027	7.8411
*C20orf118*	Chromosome 20 open reading frame 118	0.001886	1.6089	8.5961
*CD53*	CD53 molecule	0.00198	1.3513	8.7833
*C20orf118*	Chromosome 20 open reading frame 118	0.001997	1.5776	8.6481
*ATP6V0E*	ATPase, H+ transporting, lysosomal 9 kDa, V0 subunit e	0.00176	1.3477	8.9288
*ATP6V0E*	ATPase, H+ transporting, lysosomal 9 kDa, V0 subunit e	0.002317	1.3355	8.2173
*PIK3AP1*	phosphoinositide-3-kinase adaptor protein 1	0.002432	1.784	7.8028
*ADAR*	adenosine deaminase, RNA-specific	0.002492	1.3729	8.6116
*METTL7A*	methyltransferase like 7A	0.002606	−1.585	7.6029
*GCH1*	GTP cyclohydrolase 1 (dopa-responsive dystonia)	0.002631	1.6161	8.327
*SELPLG*	selectin P ligand	0.002757	1.5125	7.4685
*WSB1*	WD repeat and SOCS box-containing 1	0.00276	1.4536	8.0606
*SQRDL*	sulfide quinone reductase-like (yeast)	0.003312	1.4912	7.7882
*CXXC5*	CXXC finger 5///CXXC finger 5	0.003382	−1.6791	7.2149
FN5	...	0.013342	2.1468	6.62795
LOC441168	...	0.004813	2.3726	8.08179
SEC11L3	SEC11-like 3 (S. cerevisiae)	0.011854	−2.0503	6.92968
LOC91353	...	0.007524	−2.578	8.63641
IGLC2	immunoglobulin lambda constant 2 (Kern-Oz- marker)	0.010428	−2.6368	6.33398
TRA1	tumor rejection antigen (gp96) 1	0.005488	−1.9074	8.2753
H3F3B	H3 histone, family 3B (H3.3B)	0.018645	1.2441	9.03523
SFRS5	splicing factor, arginine/serine rish-5	0.027215	1.2557	8.35184
TMBIM4	transmembrane BAX inhibitor	0.310843	1.1017	8.58976
RHOA	ras homolog gene family	0.016069	1.1702	8.59283
XRN1	5′-3′ exoribonuclease 1	0.007922	1.3637	8.3903
SAP18	sin3-associated polypeptide, 18 kDa	0.150022	−1.1034	7.88139
ARHGEF6	Rac/Cdc42 guanine nucleotide exchange factor (GEF) 6	0.161681	1.1065	7.99103
HLA-B	major histocompatibility complex, class I, B	0.060992	1.2255	9.22158
DEK	DEK oncogene (DNA binding)	0.098052	1.1739	8.34219
EIF4A2	eukaryotic translation initiation factor 4A, isoform 2	0.27169	1.0921	8.68518

**Table 3:** List of genes shown on the “Volcano” plot according to the fold-change.

Fold-change >2			Fold-change <−2		
Symbol	*p* value	FC	Symbol	*p* value	FC
S100A6	0.019028	2.0002	MAGED1	0.001644	−2.0342
RNASET2	0.006686	2.0165	SEC11L3	0.011854	−2.0503
MT1H	0.003445	2.0475	KIAA0746	0.015464	−2.0754
S100A9	0.017515	2.0885	UBE2G1	0.003307	−2.1036
FN5	0.013342	2.1468	TPD52	0.003567	−2.2114
CD300A	0.000864	2.1544	GMNN	0.009426	−2.2142
FGR	0.009879	2.1941	SMC4L1	0.009866	−2.2396
CD97	0.007485	2.2044	UBE2J1	0.000419	−2.3147
MT1X	0.001294	2.2122	IGKV1D-13	0.011292	−2.3235
GBP1	0.004938	2.3334	PPAPDC1B	0.004347	−2.3491
CX3CR1	0.012371	2.3472	PACAP	0.016535	−2.367
TNFSF13	0.010068	2.352	PACAP	0.015153	−2.4229
LOC441168	0.004813	2.3726	LOC91353	0.007524	−2.578
SAMD9L	0.01406	2.3893	IGLC2	0.010428	−2.6368
HLA-DPB1	0.015645	2.4265	TNFRSF17	0.009406	−2.7543
SAMHD1	0.003019	2.4624	POU2AF1	0.00391	−2.7707
SERPINA1	0.00763	2.463	IGLC2	0.005057	−2.9244
GBP1	0.005006	2.4888	IGLC2	0.001172	−3.0718
FGL2	0.007811	2.6573			
DF	0.013175	2.7446			
TYROBP	0.007507	2.8639			
LOC441168	0.014525	3.2665			
EGR1	0.00334	3.2916			
FCGR3B	0.002868	4.1952			
C3AR1	0.002707	4.5817			
EGR1	0.007242	4.6155			

FC–Fold change.

**Table 4:** EASE analysis of functional overabundance in the list of top 40 DF-DHF discriminatory genes.

Immune Response Genes		Defense Response Genes		Response To Biotic Stimulus Genes		Copper/Cadmium Binding Genes		Copper Ion Homeostasis Genes	
EASE score	Bonferroni	EASE score	Bonferroni	EASE score	Bonferroni	EASE score	Bonferroni	EASE score	Bonferroni
3.91E-06	1.19E-03	1.11E-05	3.38E-03	2.26E-05	6.89E-03	3.12E-05	9.53E-03	1.52E-04	4.64E-02
CD300A		CD300A		CD300A		MT1H		MT1H	
CD97		CD97		CD97		MT1X		MT1X	
CX3CR1		CX3CR1		CX3CR1					
C3AR1		C3AR1		C3AR1					
CFD		CFD		CFD					
FCGR3B		FCGR3B		FCGR3B					
GBP1		GBP1		GBP1					
GBP2		GBP2		GBP2					
IGKV1D-13		IGKV1D-13		IGKV1D-13					
HLA-DPA1		HLA-DPA1		HLA-DPA1					
HLA-DPB1		HLA-DPB1		HLA-DPB1					
MYD88		MYD88		MYD88					
POU2AF1		POU2AF1		POU2AF1					
S100A9		S100A9		S100A9					
TNFSF12-TNFSF13		TNFSF12-TNFSF13		TNFSF12-TNFSF13					
TNFSF13		TNFSF13		TNFSF13					
TNFRSF17		TNFRSF17		TNFRSF17					
TYROBP		TYROBP		TYROBP					
		FGR							

Statistically significant overrepresented functional categories (p<0.05), after adjusting for test multiplicity.

### Identification of Classifier Genes

**Figure 5:**
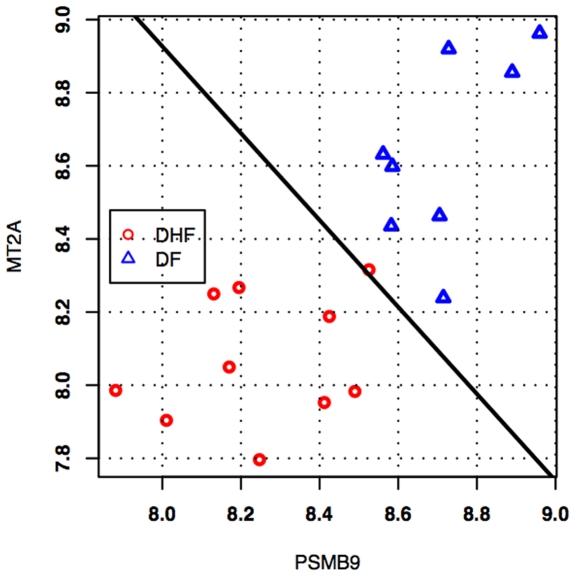
Classifier for the best pair of genes, PSMB9 and MT2A, in the discrimination of DF against DHF. Lower expression of both genes is a signature for DHF, whereas higher expression of both genes is a signature for DF. The estimated probability of error on future data for this classifier is only about 5.38%.

In addition to univariate gene selection by t-tests, we did exhaustive feature selection (all possible combinations) of single, pairs, and triplets of genes out of the prefiltered set of 1981 genes, using Linear Discrimination Analysis as the classification rule, and bolstered resubstitution as the error estimator (see Methods section). Table 5 displays the top-ten 1-gene, 2-gene and 3-gene classifiers, respectively, ranked by estimated classification error. Supplement material S6 displays top-40 1-gene and 2-gene classifiers as well as the top-100 3-gene classifiers, also ranked by estimated error. There are 37 unique genes among the top-40 pairs, while there are 86 unique genes among the top 100 triplets. The list of gene triplets is heavily dominated by the genes PSMB9, MT2A, and LOC400368. In fact, only one triplet in the top 100 does not contain any of these three genes, namely (SFRS5, PDCD4, MKNK2), which has an estimated error of 0.0383. The average estimated error for the top classifiers was as follows: 1-*gene classifier* (40) = 0.1639±0.0286, *2-gene classifier (40) = 0.0686±0.0096, 3-gene classifier (100) = 0.0395±0.0040*, which indicates that classification with pairs is more accurate than with single genes, while classification with triplets is more accurate than with pairs (the error margins above refer to a 95% confidence interval). Feature selection with two genes and three genes has the potential of “fetching” genes that cannot otherwise be found by using univariate methods (such as t-tests). This can be seen from the list of top two-gene classifiers. For example, we can see the gene for the HLA-F (which is lower expressed in DHF than in DF, data not shown). Figure 5 displays the plot of the best 2-gene classifier found by exhaustive feature selection, consisting of the pair of genes PSMB9 and MT2A. The estimated probability of error on future data for this classifier, as determined by bolstered resubstitution, is only about 5.38%. In this case, lower expression of both genes is a signature for DHF, whereas higher expression of both genes is a signature for DF.

**Table 5:** Top 10 classifiers based on either individual, duplet or triplet genes ranked by estimated classification error.

Classifiers			
Gene 1	Gene 2	Gene 3	Estimated Error
***1-Gene Classifiers***			
MT2A	-	-	0.0870
PSMB9	-	-	0.0927
IGLC2	-	-	0.1182
ADAR	-	-	0.1202
LOC400368	-	-	0.1277
FCGR3B	-	-	0.1301
HLA-F	-	-	0.1314
CD53	-	-	0.1365
VAMP3	-	-	0.1389
CXXC5	-	-	0.1443
***2-Genes Classifiers***			
PSMB9	LRRFIP1	-	0.0351
H3F3B	MT2A	-	0.0496
SFRS5	PDCD4	-	0.0501
LRRFIP1	LOC400368	-	0.0504
PSMB9	MT2A	-	0.0538
MT2A	TMBIM4	-	0.0589
HA-1	LOC400368	-	0.0602
RHOA	MT2A	-	0.0603
MT2A	XRN1	-	0.0635
MRLC2	LOC400368	-	0.0351
PSMB9	LRRFIP1	-	0.0496
H3F3B	MT2A	-	0.0501
SFRS5	PDCD4	-	0.0504
LRRFIP1	LOC400368	-	0.0538
***3-Genes Classifiers***			
HNRPA1	PSMB9	MT2A	0.0256
LRRFIP1	MRLC2	LOC400368	0.0302
PSMB9	SAP18	LRRFIP1	0.0316
PSMB9	LRRFIP1	LOC400368	0.0319
ADAR	PSMB9	ARHGEF6	0.0321
PSMB9	HLA-B	MT2A	0.0323
LRRFIP1	RPS21	LOC400368	0.0324
DEK	LRRFIP1	LOC400368	0.0326
EIF4A2	PSMB9	LRRFIP1	0.0326
DEK	ADAR	PSMB9	0.0329

### Validation of Microarray Data Using Quantitative Real Time PCR

**Figure 6:**
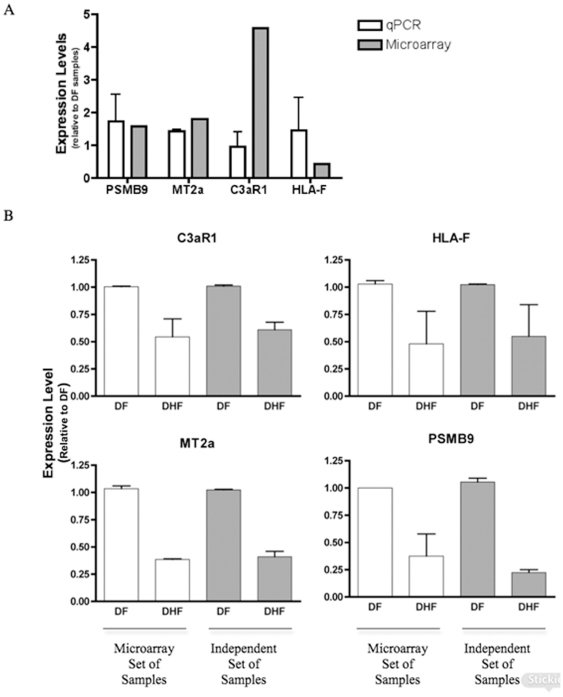
Expression levels of genes discriminating DF and DHF patients. A- For all tested genes, qPCR assays were performed using a mix of eight DF or eight DHF samples used in microarray assays. B- For all tested genes, qPCR assays were performed for a set of eight DF and eight DHF samples used in microarray assays (white solid columns), or a set of eight DF and eight DHF independent samples (grey solid columns). The experiment was performed twice and each group was analyzed in triplicates.

Quantitative real time PCR assays were performed in order to validate the results seen on the microarray assay. The following genes that were selected: PSMB9, MT2A, HLA-F and C3aR1, displayed expression levels that were predominantly increased in DF samples compared to DHF samples. Quantitative PCR of the genes cited above was performed using eight DHF and eight DF samples obtained from the same patients tested in microarray experiments. According to the Figure 6A, the qPCR quantifications showed a very good correlation with the microarray data.

In addition, qPCR was performed in a separate set of eight DHF and eight DF independent samples collected from a different set of patients from the same cohort. The qPCR results indicated that the level of expression of the genes selected was expressed at lower levels in DHF than in DF patients, in agreement with the results obtained with the 2D LDA model based on the microarray data shown in Figure 5. Hence, each of the four genes measured were expressed at lower level in DHF and they classified correctly the samples analyzed (Figure 6B). These results are very promising. We are confident that they can be the basis for a successful development of a reliable classifier to predict the clinical outcome of dengue infection.

## Discussion

A functional genomic study was performed in order to obtain insights about the early immune mechanisms associated with dengue severity and to identify biomarkers to predict the infection outcome. Initial analysis resulted in the selection of 1981 candidate genes to be statistically evaluated. The analysis of degree of similarity between the samples in a 2 and 3-dimensional spaces has indicated that the DHF group formed a very tight pattern, whereas the remaining groups were more dispersed in the plot. In the 3-dimensional MDS plot the non-dengue samples (green spheres) are grouped close together, with only one sample outlier. The DHF samples (red spheres) are all at the far right side, while the DF samples (blues spheres) are more spread apart. These observations suggest the presence of a specific pattern of gene regulation against a dengue virus infection when compared to non-specific febrile disease. Welch's two-sample t-tests were used for comparisons among the diagnostic groups: Non-Dengue vs. Dengue (DF+DHF) samples; DF vs. DHF samples and so on (Supplement material S4) but we will focus the discussion on the comparison between DHF versus DF.

The top 200 most differentially expressed genes between DF and DHF cases according to Welch's t-test was contrasted with the list of the top 200 genes according to largest fold change of average expression mRNA levels, resulting in 73 genes differentially expressed among the dengue clinical manifestations that were common to both lists. The list including all the 73 genes was further analyzed using the EASE algorithm in order to identify which categories of genes are overrepresented in this group of genes. The statistically significant overrepresented functional categories included the groups involved in immune and defense responses (Table 2). Among the nineteen genes included in the immune response category were the HLA-DPαβ genes, complement factor D, CX3CR, MyD88, TNFSF17 (BCMA), TNFSF13 (APRIL); and among the genes included in the defense response were Mixovirus resistant gene (Mx), 2′, 5′-oligoadenylate synthetase (OAS1 and OAS2) and interferon response factors (Supplement material S7), which were all less expressed in DHF. While the immune and defense response genes were expressed at lower levels in DHF, several genes associated with apoptosis responses (PDCD4, PACAP, Tumor protein D52, MAGED1, pro-apoptotic caspase adaptor protein and TNF ligand super family) were up regulated. Interestingly, CD53, a tetraspanin produced by monocytes and B cells that prevents cells from undergoing apoptosis [40] was down regulated in DHF patients, reinforcing the indication of a pro-apoptotic environment in DHF.

It is not unrealistic to expect that some of these genes could be mechanistically involved in the DHF immunopathogenesis or might be used as the basis for the prediction of dengue infection severity. Using this technology, a gene expression pattern was identified in patients suffering the most severe forms of the disease. Simmons et all [27] have shown a molecular signature on PBMCs discriminating early and late phases of DSS and they reported that genes transcripts of IFN-stimulated genes were less abundant in DSS patients than in patients without DSS, whereas the genes involved on apoptosis were up-regulated in the DSS patients. Some of the genes differentially expressed that were found by Simmons et al [27], such as MX, IFIT, pro-apoptotic caspase adaptor protein and OAS, were also found in this study. However, they were not among the most significant differentially expressed genes according to p-value in our study, perhaps because of differences on the stage and severity of the disease. In this study DHF patients were grades I or II and were compared to DF, whereas in the Simmons study the patients were DHF/DSS, grade IV and were compared with DHF patients without DSS.

In a separate study, Ubol et al [28] using samples from a cohort of children from Thailand obtained similar results associating decreased innate immune response and increased apoptosis with development of DHF. However, the individual genes identified were quite different from the ones found in this and other studies. One possible reason might be the age group used, since its known that immune response repertoire differs during early ages, where innate response predominates, as compared to adults [41]. In another interesting study, Kruif et al (2008) reported a general association of dengue severity and up regulation of genes involved on innate immune response during acute phase of infection in children [42]. However, in addition to the age group bias present in the Kruif study, the authors used RNA extracted from total leucocytes, which is composed predominantly by granulocytes, and this most have contributed for the difference in findings. This result suggests that granulocytes may be up-regulating the expression of innate immune response genes whereas the monocytic cells are suppressing it, but more detailed studies need to be done simultaneously on specific cell populations of the same patient. In summary, despite differences between the study designs and differences in the genetic background of our populations our results are consistent with the similar studies reported by Simmons, Ubol and Kruif [27], [28].

As reviewed by Clementini and Gianantonio [43], there is much evidence that genetic factors, involved on susceptibility/resistance to infectious disease, influence the immune response in humans. These factors are complex traits modulated by environmental factors, such as previous dengue infection, and do not follow Mendelian inheritance pattern. The differential expression of some of these markers are possibly due to genetic polymorphisms that can interfere with mRNA expression levels, either directly by sitting at the promoter or indirectly by interfering on the pathway that modulates the transcription of those genes. Host polymorphisms present in genes involved in dengue immune responses have been correlated with altered gene expression and susceptibility to DHF [44]. Among these genes are: the TNF-308 allele, which is associated with increased levels of TNFα, is correlated with a greater susceptibility to developing DHF [45], [46]; the wild-type *AA MBL2* genotype, which is correlated with increased levels of MBL and increased risk factors for the development of dengue-related thrombocytopenia [47] and the polymorphism of the CD209 promoter [48], which is associated with a decreased expression of DC-SIGN and possibly with a lower susceptibility of dendritic cells to be infected by the dengue virus. Thus, searching for gene expression alteration among different dengue clinical manifestation using microarray technology can suggest in a high throughput fashion, genetic factors and immunopathology mechanisms involved on dengue severity.

Moreover, during the acute phase of infection, patients suffering from DF or DHF have a very similar clinical picture. However, the disease defervescence period (after 4 to 7 days of the beginning of the symptoms) is accompanied by severity-varying circulatory disturbance signs [2]. Thus, it seems that the events preceding the defervescence may determine the outcome of the disease severity and a question of interest is the selection of a small set of the best DHF-prognosis gene markers. The MDS plot (Figure 2) including the top 40 most discriminatory genes according to p-value shows that expression patterns of DF and DHF patients are quite different and appear to be distributed into three groups; one DHF (red) group very distinct from the DF (blue) cases, and a third group, which DF and DHF are closer. This result is not surprising and it suggests that DF and DHF are extremes of a continuum spectrum of the same disease, as suggested by Sierra et al (2007), and not two separate diseases [49]. In addition, this result suggests the potential of designing a reliable classification marker based on the quantification of few gene products by qPCR or any other method to quantify RNA or protein products. Towards this goal, we selected the best multivariate sets of candidate genes for prognosis, by means of exhaustive feature selection (all possible combinations) of single, pairs and triplets of genes out of the pre-filtered set of 1981 genes, using Linear Discrimination Analysis as the classification rule, and bolstered resubstitution as the error estimator (see the Methods section). According to our results, the top 3-gene classifiers displayed an estimated rate of more than 95% chance of correct classification. We selected a few genes (PSMB9, MT2A, HLA-F and C3aR1) to test by qPCR assays. Initially, the qPCR assays were performed in the same blood samples used for the microarray study. All gene expression levels determined by qPCR were consistent with the results obtained with the microarray. Subsequently, qPCR quantification was performed in eight DF and eight DHF samples collected from an independent set of patients (Figure 6). The qPCR quantification showed that the genes (PSMB9, MT2A, HLA-F and C3aR1) were expressed at higher levels in DF than in DHF and confirmed the expression levels seen on the first set patient samples used in the microarray study and were in agreement with the 2D LDA model shown in Figure 4. Hence, each of the four genes measured were expressed at lower level in DHF and they classified correctly the samples analyzed (Figure 5B). Thus, the qPCR assay results confirmed that quantification of those genes in samples collected on the first medical visit of a dengue infected patient can be used to predict whether the individual will develop DHF symptoms two or three days later.

Our data indicates that the classification of patients into DF and DHF on the basis of gene profiling is feasible and may be a useful means of guiding clinical management of dengue patients. Further analyses using additional independent samples are underway to confirm the value of these classifiers. However, some points need to be addressed on future studies, including the validation of the gene markers identified here on samples collected from people infected with other dengue serotypes for ultimately support the development of a qPCR-based kit to predict the clinical outcome of people infected with any of the dengue serotypes during the first days of the symptoms. Besides the patient-management benefits, this study can also help on the characterization of natural dengue infection and hopefully will facilitate the elucidation of the molecular mechanisms involved in DHF.

## Supporting Information

Material S1 Noise and efficiency measurements. Arrows indicate the only array, in the non-dengue group,which showed a reduced signal/noise ratio and percentage of present calls.
(4.70 MB TIF)

Material S2 RNA degradation plot.
(1.47 MB TIF)

Material S3 Volcano plots showing p-values correlated to fold changes in four different comparisons: D (DF+DHF) vs. ND (A), DF vs. ND (B), DHF vs. ND (C) and DF vs DHF (D).
(4.20 MB TIF)

Material S4 The top 40 genes with the most significant p-values among the 1981 genes selected, for each of the four comparisons (D (DF+DHF) vs. ND, DF vs. ND, DHF vs. ND and DF vs DHF), along with fold change values, average signal intensity, and annotation from the DAVID Bioinformatics Database at NIAID/NIH (http://david.abcc.ncifcrf.gov/).
(0.11 MB XLS)

Material S5 The EASE results for all 4 comparisons (D (DF+DHF) vs. ND, DF vs. ND, DHF vs. ND and DF vs DHF).
(0.03 MB XLS)

Material S6 Classifiers based on either individual, duplet or triplet genes ranked by estimated classification error.
(0.17 MB DOC)

Material S7 Genes included in the immune and defense responses after analysis using the EASE algoritm.
(0.05 MB DOC)
