# Supplementary figures and images for: Gene Expression Profiling during Early Acute Febrile Stage of Dengue Infection Can Predict the Disease Outcome
Source: PLoS One. 2009 Nov 19;4(11):e7892. doi: 10.1371/journal.pone.0007892 (PMC2775946; doi:10.1371/journal.pone.0007892)

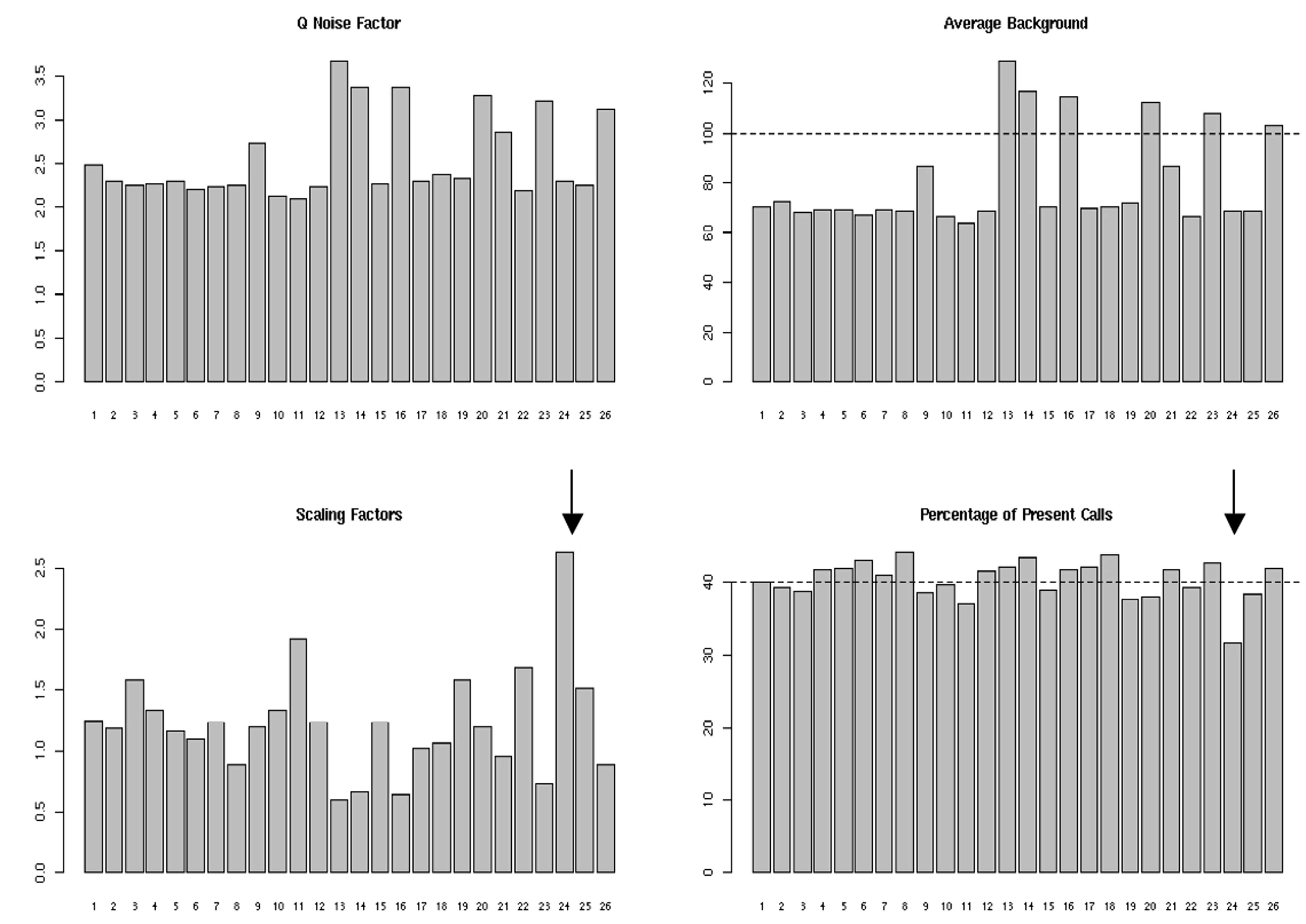

Supplement: Material S1 — Noise and efficiency measurements. Arrows indicate the only array, in the non-dengue group,which showed a reduced signal/noise ratio and percentage of present calls. (4.70 MB TIF) [file pone.0007892.s001.tif]

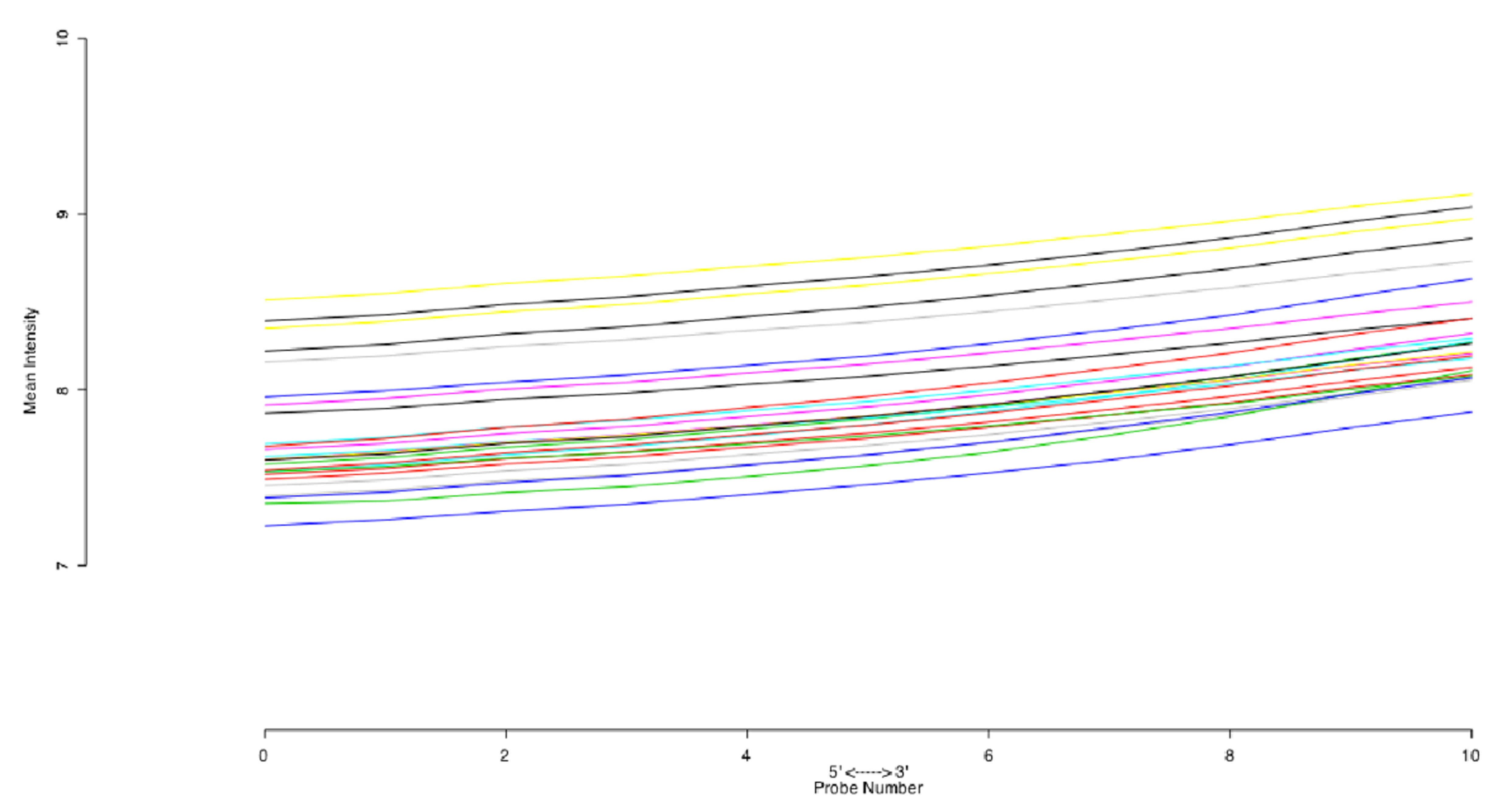

Supplement: Material S2 — RNA degradation plot. (1.47 MB TIF) [file pone.0007892.s002.tif]

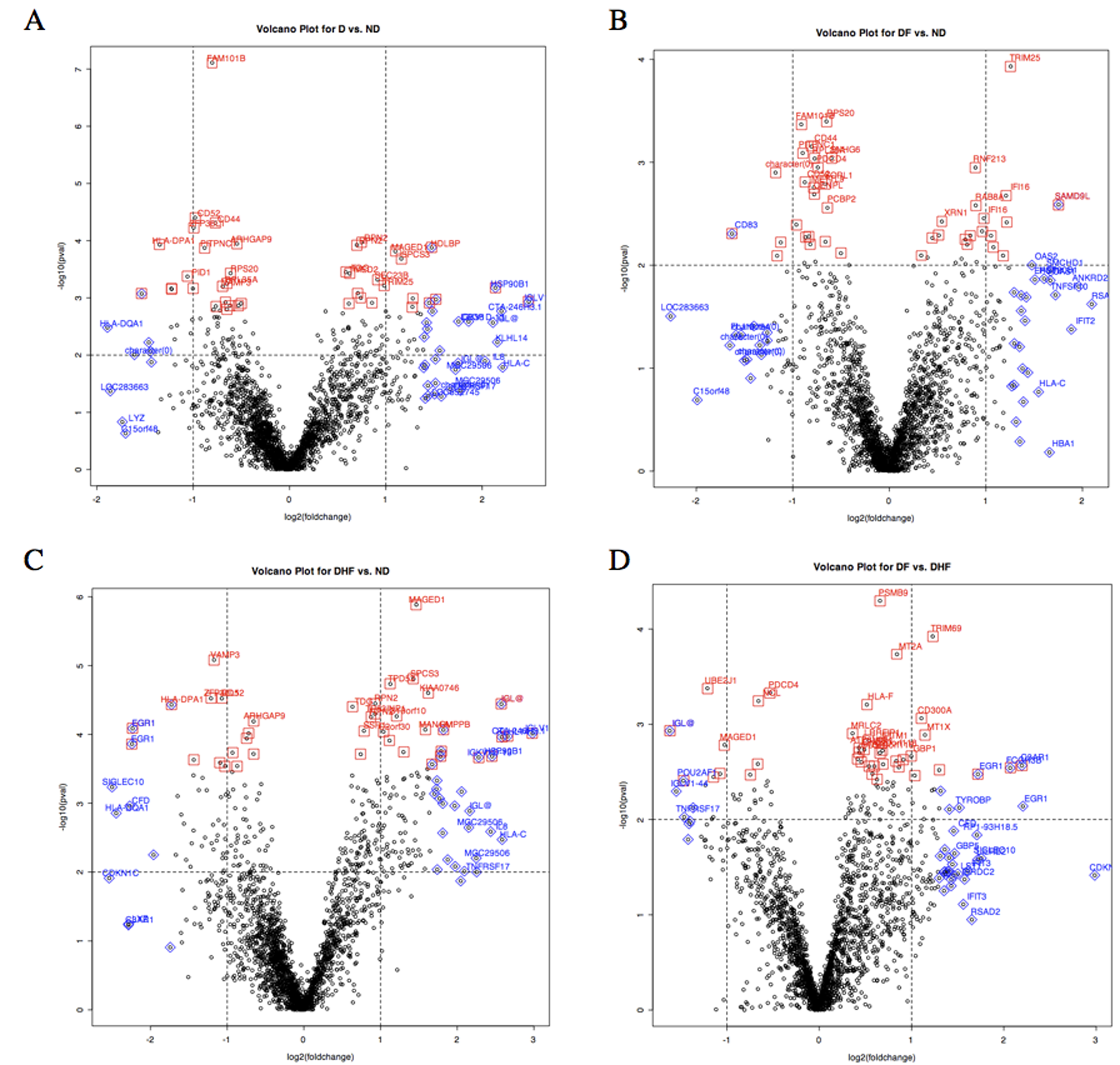

Supplement: Material S3 — Volcano plots showing p-values correlated to fold changes in four different comparisons: D (DF+DHF) vs. ND (A), DF vs. ND (B), DHF vs. ND (C) and DF vs DHF (D). (4.20 MB TIF) [file pone.0007892.s003.tif]
