# Supplementary material for: Gene Expression Profiling during Early Acute Febrile Stage of Dengue Infection Can Predict the Disease Outcome
Source: PLoS One. 2009 Nov 19;4(11):e7892. doi: 10.1371/journal.pone.0007892 (PMC2775946; doi:10.1371/journal.pone.0007892)
Supplement: Material S6 — Classifiers based on either individual, duplet or triplet genes ranked by estimated classification error. (0.17 MB DOC) [file pone.0007892.s006.doc]

**Supplement material S6.**

| **Gene 1** | **Gene 2** | **Gene 3** | **Estimated Error** |
| --- | --- | --- | --- |
| ***1-Gene Classifiers*** |  |  |  |
| MT2A | - | - | 0.0870 |
| PSMB9 | - | - | 0.0927 |
| IGLC2 | - | - | 0.1182 |
| ADAR | - | - | 0.1202 |
| LOC400368 | - | - | 0.1277 |
| FCGR3B | - | - | 0.1301 |
| HLA-F | - | - | 0.1314 |
| CD53 | - | - | 0.1365 |
| VAMP3 | - | - | 0.1389 |
| CXXC5 | - | - | 0.1443 |
| DKFZP586 | - | - | 0.1477 |
| LOC91353 | - | - | 0.1513 |
| LRRFIP1 | - | - | 0.1555 |
| MT1H | - | - | 0.1604 |
| SAMHD1 | - | - | 0.1626 |
| UBE2J1 | - | - | 0.1634 |
| PDCD4 | - | - | 0.1669 |
| PACAP | - | - | 0.1674 |
| POU2AF1 | - | - | 0.1688 |
| KLHL14 | - | - | 0.1695 |
| FYB | - | - | 0.1708 |
| PACAP | - | - | 0.1729 |
| (no symbol) | - | - | 0.1734 |
| SEC11L3 | - | - | 0.1749 |
| NDUFB6 | - | - | 0.1767 |
| MT1X | - | - | 0.1772 |
| NCL | - | - | 0.1793 |
| C1QBP | - | - | 0.1821 |
| HIST1H4C | - | - | 0.1850 |
| SAMHD1 | - | - | 0.1881 |
| TNFRSF17 | - | - | 0.1885 |
| IFITM1 | - | - | 0.1893 |
| IGKC | - | - | 0.1910 |
| CHSY1 | - | - | 0.1920 |
| MRLC2 | - | - | 0.1933 |
| CXXC5 | - | - | 0.1941 |
| LOC400741 | - | - | 0.1956 |
| ATP6V0E | - | - | 0.1963 |
| HSPA5 | - | - | 0.1966 |
| TPD52 | - | - | 0.1970 |
| ***2-Genes Classifiers*** |  |  |  |
| PSMB9 | LRRFIP1 | - | 0.0351 |
| H3F3B | MT2A | - | 0.0496 |
| SFRS5 | PDCD4 | - | 0.0501 |
| LRRFIP1 | LOC400368 | - | 0.0504 |
| PSMB9 | MT2A | - | 0.0538 |
| MT2A | TMBIM4 | - | 0.0589 |
| HA-1 | LOC400368 | - | 0.0602 |
| RHOA | MT2A | - | 0.0603 |
| MT2A | XRN1 | - | 0.0635 |
| MRLC2 | LOC400368 | - | 0.0661 |
| NCL | PSMB9 | - | 0.0665 |
| DKFZP586 | LOC400368 | - | 0.0666 |
| MT2A | CNOT2 | - | 0.0669 |
| ITM2B | LOC400368 | - | 0.0687 |
| PSMB9 | HA-1 | - | 0.0688 |
| CD52 | LOC400368 | - | 0.0702 |
| ADAR | MT2A | - | 0.0708 |
| MT2A | HIPK3 | - | 0.0709 |
| SELL | MT2A | - | 0.0710 |
| PSMB9 | ATP6V0E | - | 0.0713 |
| PSMB9 | MRLC2 | - | 0.0715 |
| NCL | MT2A | - | 0.0721 |
| RAP1B | MT2A | - | 0.0730 |
| MT2A | LOC400368 | - | 0.0733 |
| PSMB9 | HLA-C | - | 0.0736 |
| ADAR | PSMB9 | - | 0.0740 |
| PSMB9 | FNBP3 | - | 0.0742 |
| MT2A | ACTR3 | - | 0.0743 |
| PSMB9 | ARHGEF6 | - | 0.0744 |
| UBE2J1 | LOC400368 | - | 0.0752 |
| SFRS5 | MT2A | - | 0.0762 |
| RPS25 | PSMB9 | - | 0.0765 |
| TMSB4X | LOC400368 | - | 0.0766 |
| PSMB9 | RPL14 | - | 0.0766 |
| RPS15A | LOC400368 | - | 0.0769 |
| PSMB9 | GNA13 | - | 0.0769 |
| EIF5A | LOC400368 | - | 0.0769 |
| ARPC1B | BTN3A3 | - | 0.0770 |
| PSMB9 | MYL6 | - | 0.0772 |
| RPL9 | LOC400368 | - | 0.0773 |
| ***3-Genes Classifiers*** |  |  |  |
| HNRPA1 | PSMB9 | MT2A | 0.0256 |
| LRRFIP1 | MRLC2 | LOC400368 | 0.0302 |
| PSMB9 | SAP18 | LRRFIP1 | 0.0316 |
| PSMB9 | LRRFIP1 | LOC400368 | 0.0319 |
| ADAR | PSMB9 | ARHGEF6 | 0.0321 |
| PSMB9 | HLA-B | MT2A | 0.0323 |
| LRRFIP1 | RPS21 | LOC400368 | 0.0324 |
| DEK | LRRFIP1 | LOC400368 | 0.0326 |
| EIF4A2 | PSMB9 | LRRFIP1 | 0.0326 |
| DEK | ADAR | PSMB9 | 0.0329 |
| PSMB9 | HLA-C | MT2A | 0.0331 |
| MT2A | ACTB | LOC400368 | 0.0332 |
| CAP1 | LRRFIP1 | LOC400368 | 0.0339 |
| PSMB9 | MT2A | HLA-C | 0.0339 |
| PSMB9 | MT2A | HNRPA1 | 0.0340 |
| PSMB9 | MT2A | RPL14 | 0.0344 |
| PSMB9 | DDX17 | MT2A | 0.0344 |
| PSMB9 | MT2A | HLA-A | 0.0353 |
| MT2A | ARHGEF10 | LOC400368 | 0.0353 |
| HNRPA1 | MT2A | LOC400368 | 0.0353 |
| ADAR | PSMB9 | LRRFIP1 | 0.0358 |
| HLA-E | PSMB9 | MT2A | 0.0362 |
| PSMB9 | MT2A | EEF1D | 0.0362 |
| LRRFIP1 | ITM2B | LOC400368 | 0.0364 |
| PSMC1 | PSMB9 | LRRFIP1 | 0.0367 |
| LRRFIP1 | PTMA | LOC400368 | 0.0374 |
| SKP1A | LRRFIP1 | LOC400368 | 0.0374 |
| PSMB9 | H3F3B | MT2A | 0.0378 |
| PSMB9 | MT2A | PTPRC | 0.0381 |
| LRRFIP1 | TMSB4X | LOC400368 | 0.0382 |
| SFRS5 | PDCD4 | MKNK2 | 0.0383 |
| PSMB9 | MT2A | RAC2 | 0.0384 |
| PSMB1 | PSMB9 | LRRFIP1 | 0.0385 |
| DEK | PSMB9 | LRRFIP1 | 0.0389 |
| LRRFIP1 | MRFAP1 | LOC400368 | 0.0389 |
| LRRFIP1 | ARPC2 | LOC400368 | 0.0390 |
| EIF4A2 | LRRFIP1 | LOC400368 | 0.0392 |
| PSMB9 | DDX17 | LRRFIP1 | 0.0394 |
| PSMB9 | MT2A | MRLC2 | 0.0395 |
| ADAR | PSMB9 | MT2A | 0.0397 |
| PSMB9 | MT2A | RPS19 | 0.0397 |
| MT2A | HNRPA1 | LOC400368 | 0.0402 |
| TMEM66 | PSMB9 | MT2A | 0.0402 |
| LRRFIP1 | GLTSCR2 | LOC400368 | 0.0403 |
| PSMB9 | MT2A | HLA-A | 0.0403 |
| LRRFIP1 | CD47 | LOC400368 | 0.0403 |
| LRRFIP1 | KPNB1 | LOC400368 | 0.0404 |
| PSMB9 | SNX3 | MT2A | 0.0404 |
| PSMB9 | LRRFIP1 | C1orf43 | 0.0405 |
| UBE2J1 | IGKC | LOC400368 | 0.0406 |
| PSMB9 | LRRFIP1 | IFITM1 | 0.0407 |
| PSMB9 | BTN3A3 | LRRFIP1 | 0.0408 |
| UCP2 | LRRFIP1 | LOC400368 | 0.0408 |
| SAP18 | LRRFIP1 | LOC400368 | 0.0411 |
| LRRFIP1 | HA-1 | LOC400368 | 0.0411 |
| HNRPA1 | HA-1 | LOC400368 | 0.0411 |
| UBE2J1 | IGKC | LOC400368 | 0.0411 |
| NAP1L1 | LRRFIP1 | LOC400368 | 0.0412 |
| LRRFIP1 | MORF4L1 | LOC400368 | 0.0413 |
| LRRFIP1 | MT2A | LOC400368 | 0.0413 |
| ARL6IP5 | LRRFIP1 | LOC400368 | 0.0415 |
| LRRFIP1 | GGA1 | LOC400368 | 0.0416 |
| STK24 | LRRFIP1 | LOC400368 | 0.0416 |
| PSMB10 | LRRFIP1 | LOC400368 | 0.0417 |
| PSMB9 | EEF1A1 | MT2A | 0.0417 |
| PSMB9 | MT2A | LOC440055 | 0.0418 |
| RPL35 | LRRFIP1 | LOC400368 | 0.0420 |
| --- | LRRFIP1 | LOC400368 | 0.0420 |
| RPS29 | PSMB9 | MT2A | 0.0420 |
| MT2A | RPL14 | LOC400368 | 0.0423 |
| ACTB | MT2A | LOC400368 | 0.0425 |
| RPS27 | PSMB9 | MT2A | 0.0425 |
| LRRFIP1 | H3F3B | LOC400368 | 0.0425 |
| HA-1 | HNRPA1 | LOC400368 | 0.0425 |
| NCL | MT2A | RAC2 | 0.0427 |
| ARPC1B | PSMB9 | BTN3A3 | 0.0428 |
| LRRFIP1 | PSMB1 | LOC400368 | 0.0429 |
| TOMM7 | LRRFIP1 | LOC400368 | 0.0429 |
| MT2A | HLA-A | LOC400368 | 0.0430 |
| COX4I1 | LRRFIP1 | LOC400368 | 0.0430 |
| CAP1 | ADAR | PSMB9 | 0.0430 |
| LRRFIP1 | SEPT9 | LOC400368 | 0.0431 |
| --- | UBE2J1 | LOC400368 | 0.0431 |
| PSMB9 | LRRFIP1 | --- | 0.0432 |
| TXNL4A | PSMB9 | LRRFIP1 | 0.0433 |
| --- | UBE2J1 | LOC400368 | 0.0433 |
| --- | UBE2J1 | LOC400368 | 0.0434 |
| ADAR | PSMB9 | CD52 | 0.0435 |
| NCL | RAP1B | MT2A | 0.0435 |
| TPD52 | IGKC | LOC400368 | 0.0436 |
| ACTB | MT2A | LOC400368 | 0.0436 |
| EIF4A2 | PSMB9 | MT2A | 0.0436 |
| LRRFIP1 | YWHAB | LOC400368 | 0.0436 |
| PTMA | LRRFIP1 | LOC400368 | 0.0437 |
| HLA-B | MT2A | LOC400368 | 0.0438 |
| PSMB9 | LRRFIP1 | KPNB1 | 0.0439 |
| LRRFIP1 | TMSB10 | LOC400368 | 0.0439 |
| LAMP1 | PSMB9 | MT2A | 0.0440 |
| LRRFIP1 | --- | LOC400368 | 0.0440 |
| MT2A | DAZAP2 | LOC400368 | 0.0440 |
| HNRPA1 | PSMB9 | MT2A | 0.0256 |
| LRRFIP1 | MRLC2 | LOC400368 | 0.0302 |
