# Supplementary material for: Gene Expression Profiling during Early Acute Febrile Stage of Dengue Infection Can Predict the Disease Outcome
Source: PLoS One. 2009 Nov 19;4(11):e7892. doi: 10.1371/journal.pone.0007892 (PMC2775946; doi:10.1371/journal.pone.0007892)
Supplement: Material S7 — Genes included in the immune and defense responses after analysis using the EASE algoritm. (0.05 MB DOC) [file pone.0007892.s007.doc]

**Supplement Material S7**.

| **Functional Category**  (Genes) | **GenBank** | **Description** |
| --- | --- | --- |
| ***Immune Response*** | | |
| HLA-DPB1 | NM_002121 | major histocompatibility complex, c |
| GBP1 | BC002666 | guanylate binding protein 1, interf |
| GBP1 | NM_002053 | guanylate binding protein 1, interf |
| GBP2 | NM_004120 | guanylate binding protein 2, interf |
| CD97 | NM_001784 | CD97 molecule |
| S100A9 | NM_002965 | S100 calcium binding protein A9 |
| FCGR3B | J04162 | Fc fragment of IgG, low affinity II |
| TYROBP | NM_003332 | TYRO protein tyrosine kinase bindin |
| POU2AF1 | NM_006235 | POU class 2 associating factor 1 |
| CFD | NM_001928 | complement factor D (adipsin) |
| CX3CR1 | U20350 | chemokine (C-X3-C motif) receptor 1 |
| TNFRSF17 | NM_001192 | tumor necrosis factor receptor supe |
| MYD88 | U70451 | myeloid differentiation primary res |
| C3AR1 | U62027 | complement component 3a receptor 1 |
| CD300A | AF020314 | CD300a molecule |
| TNFSF13 | AF114013 | tumor necrosis factor (ligand) supe |
| HLA-DPA1 | M27487 | major histocompatibility complex, c |
| IGKV1D-13 | AW408194 | immunoglobulin kappa variable 1D-13 |
| GBP1 | AW014593 | guanylate binding protein 1, interf |
| ***Defense Response*** | | |
| MX1 | NM_002462 | myxovírus (influenza vírus) resista |
| OAS1 | NM_016816 | 2',5'-oligoadenylate synthetase 1, |
| OAS2 | NM_016817 | 2'-5'-oligoadenylate synthetase 2, |
| IFI16 | NM_005531 | interferon, gamma-inducible protein |
| IFI16 | AF208043 | interferon, gamma-inducible protein |
| IFI35 | BC001356 | interferon-induced protein 35 |
| IFI44 | NM_006417 | interferon-induced protein 44 |
